# Intrafamilial Phenotypic Variability in Tuberous Sclerosis Complex: Emphasis on Oral and Radiographic Findings in Two Siblings

**DOI:** 10.7759/cureus.111022

**Published:** 2026-06-17

**Authors:** Rashmi Yadav, Guru R Prasad, Akshay Kumar

**Affiliations:** 1 Oral Medicine and Radiology, H.P. Government Dental College, Shimla, IND; 2 Radiodiagnosis, Indira Gandhi Medical College, Shimla, IND

**Keywords:** enamel pits, gingival fibroma, intrafamilial variability, pulpal calcifications, subependymal nodules, tuberous sclerosis complex

## Abstract

Tuberous sclerosis complex (TSC) is an autosomal dominant multisystem disorder distinguished by hamartomatous lesions involving multiple organs, most notably the brain, skin, kidneys, and oral cavity. Considerable variability in clinical expression is often observed even among affected members of the same family. We describe two siblings, aged 12 and 8 years, who presented for dental evaluation and were found to have systemic and orofacial features consistent with TSC. Both children displayed multiple cutaneous manifestations and demonstrated subependymal nodules on magnetic resonance imaging of the brain, yet neither exhibited seizures or other neurological symptoms, in contrast to their father, who had a documented seizure history. The elder sibling presented with multiple enamel pits, three sessile gingival fibromas, and radiographic evidence of pulpal calcifications, whereas the younger sibling exhibited only a few enamel pits without other oral features. This report illustrates the heterogeneity of clinical expression that may exist within a single family and underscores the value of a thorough oral and radiographic evaluation in establishing a diagnosis of TSC.

## Introduction

Tuberous sclerosis complex (TSC), also known as Bourneville’s disease, is a rare autosomal-dominant neurocutaneous syndrome [1]. It was first described in 1880 by Désiré-Magloire Bourneville. It is a rare genetic disorder affecting both sexes and all ethnic groups [2]. The condition affects one in every 6,000 to 10,000 individuals [3]. Mutations in two genetic loci have been identified as the underlying pathogenic mechanism: TSC-1 (chromosome 9) encoding hamartin, and TSC-2 (chromosome 16) encoding tuberin [4]. Hamartin forms a complex with tuberin, which inhibits cellular signaling through mTOR and acts as a negative regulator of the cell cycle [5]. Familial cases account for 50% of cases and are mostly due to the TSC1 gene mutation. Some individuals acquire TSC through a process called gonadal mosaicism [6]. Early diagnosis and intervention are essential for improving patient prognosis, as symptoms may remain latent until later stages. Currently, the diagnosis of TSC can be achieved through genetic testing or clinical evaluation, although approximately 10% to 15% of patients may lack identifiable mutations [7].

The diagnosis of TSC is based on established clinical criteria, including a combination of major and minor features such as hypomelanotic macules, facial angiofibromas, shagreen patches, subependymal nodules, and characteristic oral manifestations including enamel pits and gingival fibromas. According to the updated International Tuberous Sclerosis Complex Consensus Conference recommendations, a definite diagnosis is established when specific combinations of these features are present or when a pathogenic TSC1 or TSC2 mutation is identified [8]. Given that oral findings may represent early or readily detectable manifestations of the disease, dental professionals can play an important role in the recognition and multidisciplinary management of affected individuals.

## Case presentation

Family history of the patients

The father was a known case of tuberous sclerosis with a history of seizures. Documentary medical records were unavailable. The mother had no contributory history.

Case 1: 12-year-old female

A 12-year-old female sibling presented for dental evaluation. General physical examination revealed multiple cutaneous manifestations, including multiple facial angiofibromas (Figure 1), more than three hypomelanotic macules, and a shagreen patch over the facial region (Figure 2).

**Figure 1:**
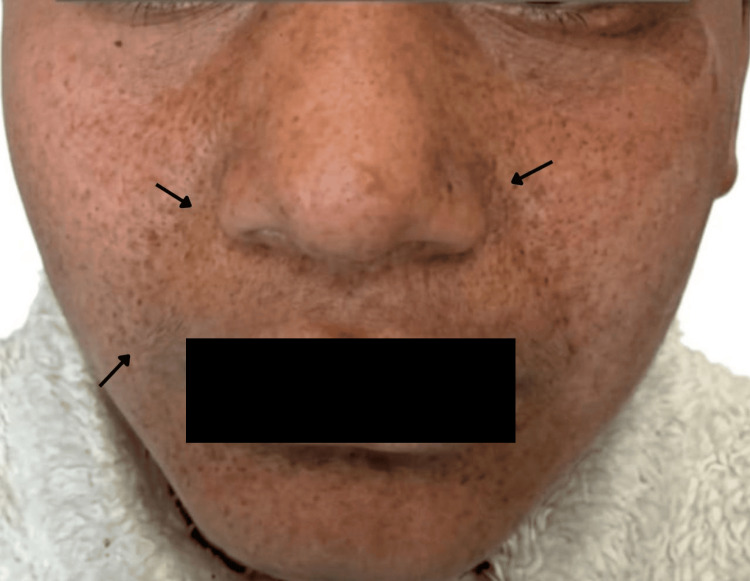
Facial angiofibromas in case 1 patient with TSC Multiple erythematous to skin-coloured, small papular lesions are seen distributed symmetrically over the bilateral malar regions, nasal dorsum, nasolabial folds, and perioral region, sparing the upper lip. The lesions are consistent with facial angiofibromas (adenoma sebaceum), a characteristic cutaneous manifestation of tuberous sclerosis complex (TSC). Note the butterfly-like distribution centered over the nose and cheeks.

**Figure 2:**
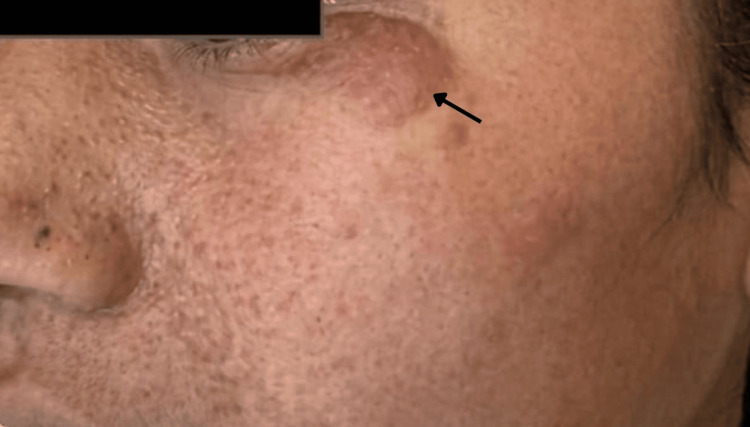
A shagreen patch in case 1 patient with TSC A well-demarcated, slightly raised, brownish-pigmented plaque with a characteristic cobblestone or "orange-peel" surface texture is seen lateral and inferior to the left eye (indicated by an arrow). The lesion displays the typical collagenomatous, firm, irregularly nodular surface consistent with a shagreen patch (connective tissue nevus), a pathognomonic cutaneous feature of tuberous sclerosis complex (TSC). The background skin also demonstrates scattered facial angiofibromas.

There was no history of seizures, cognitive impairment, or other neurological complaints. Intraoral examination showed multiple enamel pits involving teeth 12, 21, 33, 43, and 44. Additionally, three firm, sessile, non-tender gingival fibromas were observed in the anterior marginal gingiva on the labial aspect. Based on the characteristic presentation in a patient with TSC, a clinical diagnosis of gingival fibromas was made. Histopathological examination was not performed as the lesions were asymptomatic (Figure 3).

**Figure 3:**
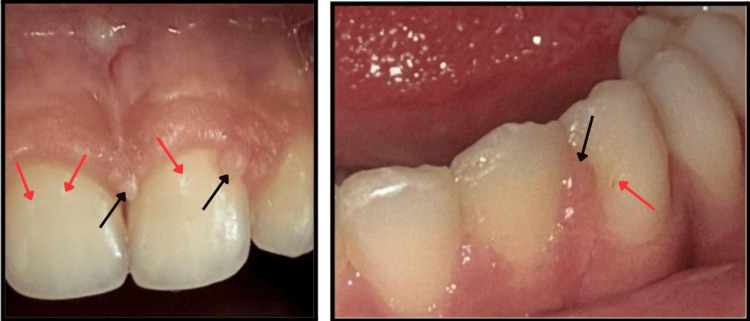
Intraoral photograph of anterior teeth of case 1 patient The images show multiple enamel pits on the labial surfaces (red arrows) and adjacent gingival fibromas presenting as small, sessile, nodular overgrowths along the gingival margin (black arrows).

Radiographic evaluation (IOPA and panoramic radiograph) revealed multiple discrete radiopaque masses within the pulp chambers of anterior and posterior teeth, suggestive of pulpal calcifications, with no associated periapical pathology (Figures 4, 5).

**Figure 4:**
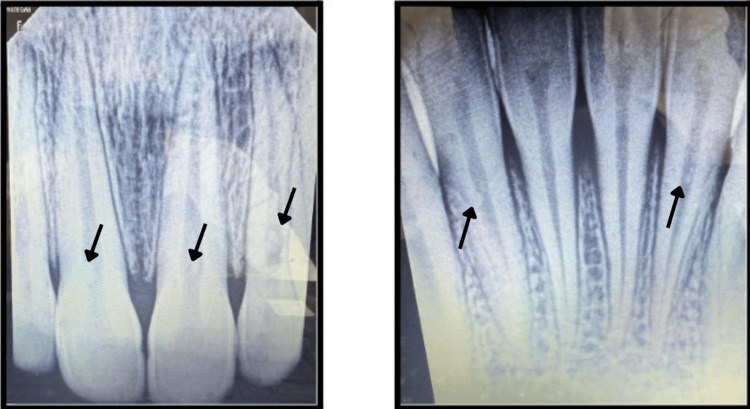
Intraoral periapical radiographs (IOPA) of maxillary and mandibular anterior teeth of case 1 patient The images demonstrate intrapulpal calcifications (black arrows), seen as well-defined radiopaque masses within the pulp chambers and root canals.

**Figure 5:**
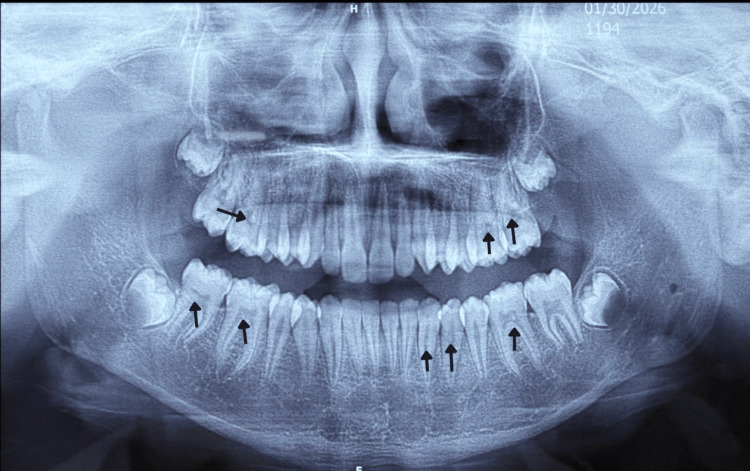
Orthopantomogram (OPG) image of case 1 patient. The image demonstrates multiple intrapulpal calcifications (black arrows) involving both maxillary and mandibular dentition. These are seen as discrete radiopaque foci within the pulp chambers and root canals of several teeth, with no evident associated periapical pathology.

Magnetic resonance imaging (MRI) of the brain demonstrated multiple subependymal nodules along the lateral ventricular walls and cortical nodules in the bilateral fronto-parietal lobes, consistent with features of TSC (Figure 6).

**Figure 6:**
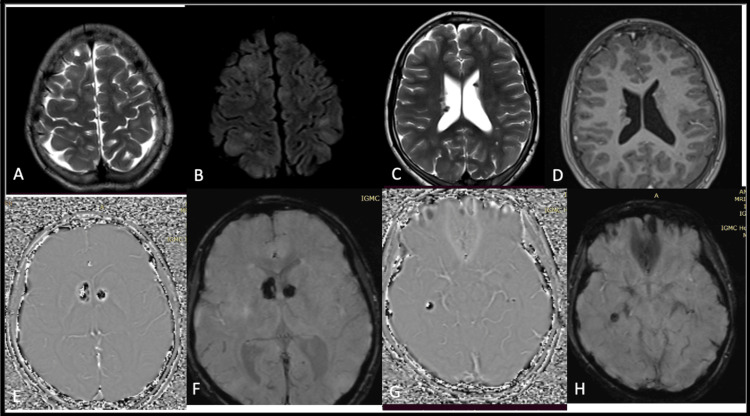
T2-FLAIR MRI (axial) images of case 1 patient's brain The images show multiple subcortical hyperintense lesions in the bilateral fronto-parietal lobes (A, B); T2 hypointense and T1 hyperintense subependymal nodule(C, D) showing blooming on phase and magnitude images (E-H) suggestive of calcification. T2-FLAIR: T2-weighted Fluid-Attenuated Inversion Recovery.

Treatment and Management

Management was directed toward preventive dental care and coordination of systemic surveillance. At the initial visit, the patient and her parents received reinforced oral hygiene instruction, dietary counseling on limiting refined carbohydrates, and a professional dental prophylaxis. Topical 5% sodium fluoride varnish was applied to all enamel-pit-affected teeth, and resin-based pit-and-fissure sealants were subsequently placed over the defects to occlude the porous enamel and reduce future caries risk. Because the three gingival fibromas were small, sessile, asymptomatic, and did not interfere with function or aesthetics, a conservative non-surgical approach was elected, with surgical excision reserved for any subsequent enlargement, ulceration, or functional compromise. The pulpal calcifications were asymptomatic and incidental; no endodontic intervention was undertaken, but the findings were documented for future reference should restorative or endodontic treatment become necessary.

In parallel with dental management, the patient was referred for multidisciplinary evaluation in keeping with the 2021 international TSC surveillance recommendations [8]. Referrals were initiated to paediatric neurology for serial assessment of the subependymal nodules and screening for subependymal giant cell astrocytoma; to dermatology for evaluation and longitudinal management of the facial angiofibromas, hypomelanotic macules, and shagreen patch; to ophthalmology for retinal examination; to nephrology with abdominal ultrasonography to screen for renal angiomyolipomas; and to clinical genetics for counselling of the family regarding the autosomal dominant inheritance pattern and reproductive implications.

Response and Follow-up

The patient tolerated the dental procedures well, with no immediate complications. At the three-month review, the placed sealants were intact, oral hygiene had improved, and no new carious lesions or symptoms were identified. The gingival fibromas remained stable in size, color, and consistency, and the patient reported no functional or aesthetic concerns. The patient has been enrolled in a long-term recall program with dental review every six months and concurrent multidisciplinary surveillance in line with consensus guidelines.

Case 2: eight-year-old male

An eight-year-old male patient presented for routine dental evaluation. General physical examination revealed multiple cutaneous manifestations, including multiple facial angiofibromas (Figure 7), one hypomelanotic macule and confetti skin lesions (Figure 8), and two ungual fibromas (Figure 9).

**Figure 7:**
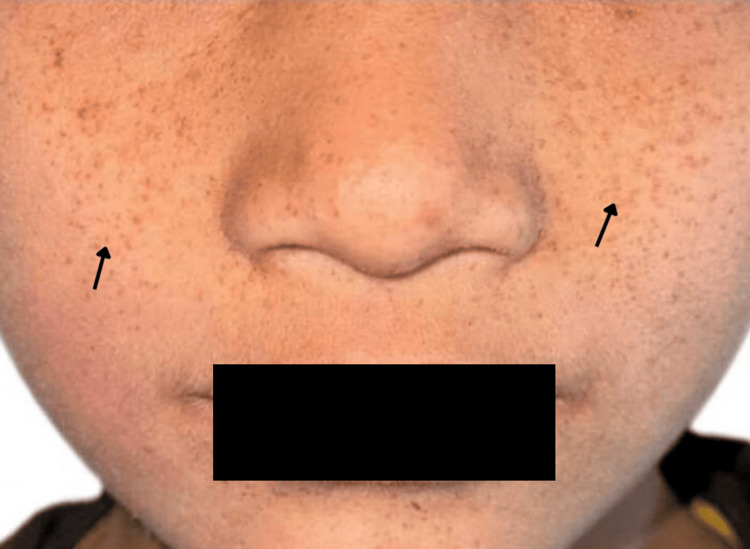
Extraoral photograph of case 2 patient The image shows multiple facial angiofibromas distributed symmetrically over the malar region and nose, presenting as numerous small, reddish-brown papular lesions characteristic of the condition.

**Figure 8:**
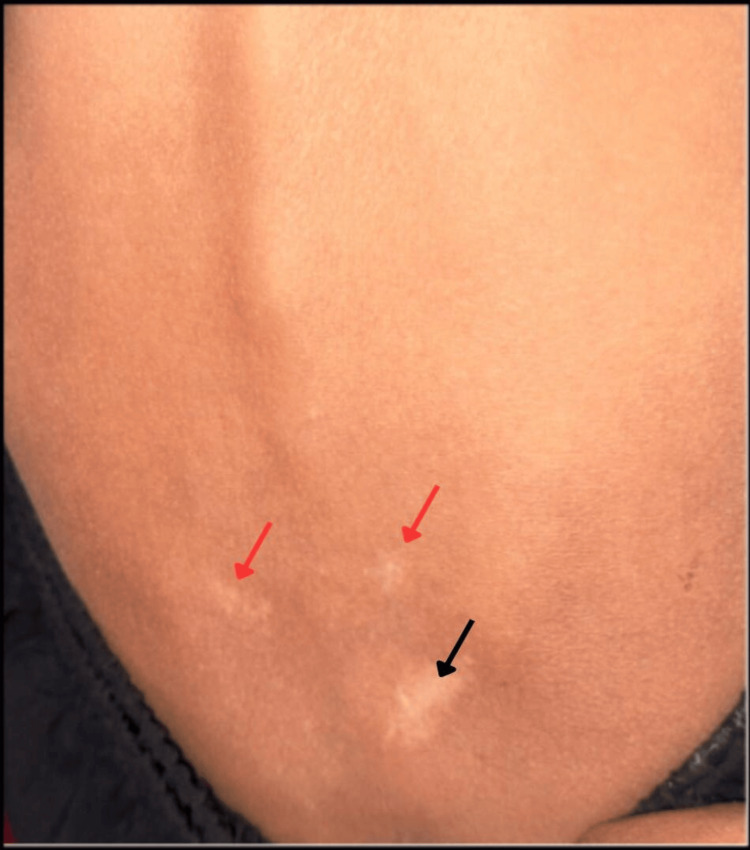
Clinical photograph of case 2 patient's back The image shows cutaneous findings over the lumbosacral region. The black arrow indicates a well-defined hypomelanotic macule (ash-leaf macule), and the red arrow highlights multiple small, scattered hypopigmented spots representing "confetti" skin lesions.

**Figure 9:**
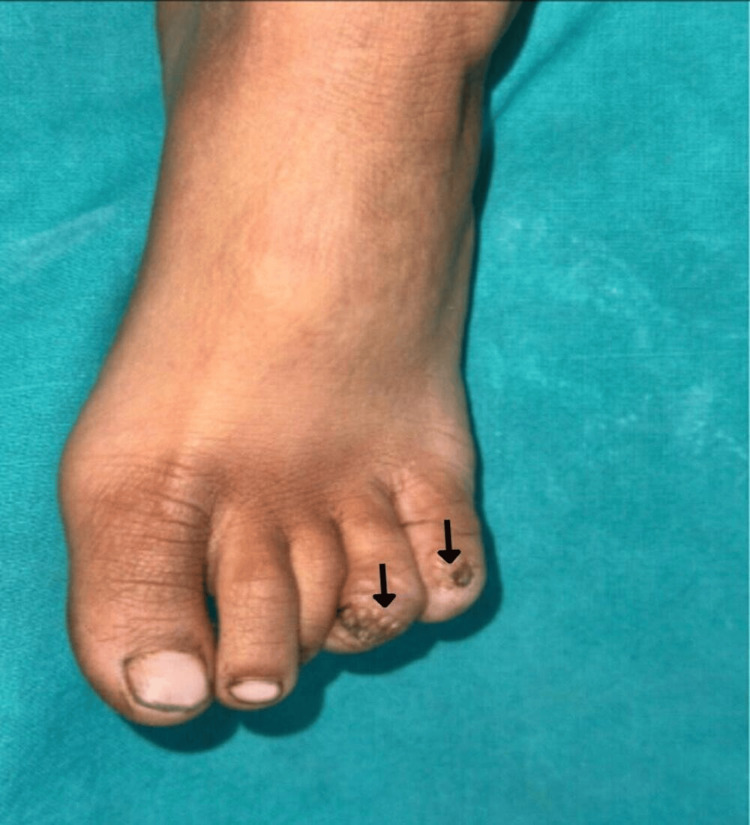
Clinical photograph of case 2 patient's left foot The image shows periungual/subungual fibromas (Koenen's tumors). The black arrows indicate firm, nodular growths arising from the nail folds of the third and fourth toes, with associated nail plate dystrophy and longitudinal grooving. These ungual fibromas are a major diagnostic criterion for Tuberous Sclerosis Complex (TSC).

There was no history of seizures, developmental delay, or other neurological complaints. Intraoral examination demonstrated enamel pits involving teeth 11 and 21 (Figure 10) with no gingival fibromas observed intra orally (Figure 11).

**Figure 10:**
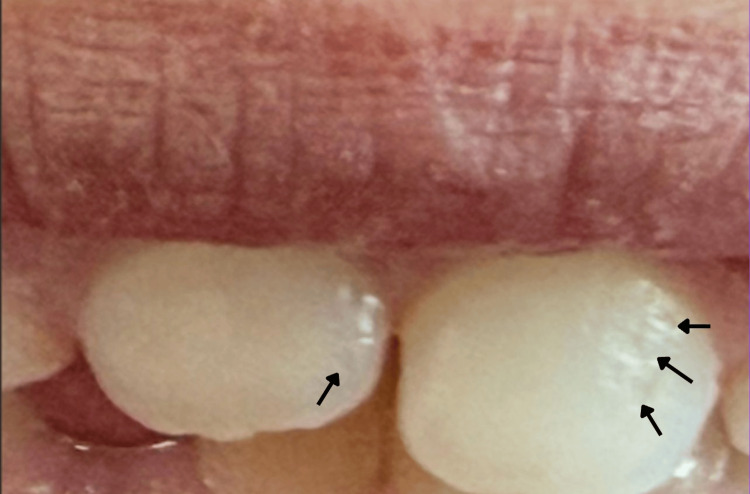
Intraoral clinical photograph of case 2 patient Black arrows in the image show multiple dental enamel pits on the labial surfaces of the maxillary anterior teeth.

**Figure 11:**
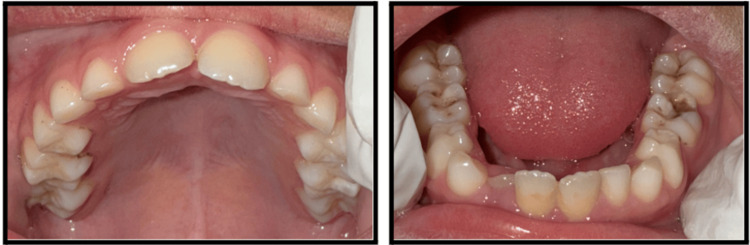
Case 2, Intra oral examination revealed no presentation of gingival fibromas

Panoramic radiograph (OPG) did not reveal any evidence of pulpal calcifications, jaw lesions, or periapical pathology (Figure 12).

**Figure 12:**
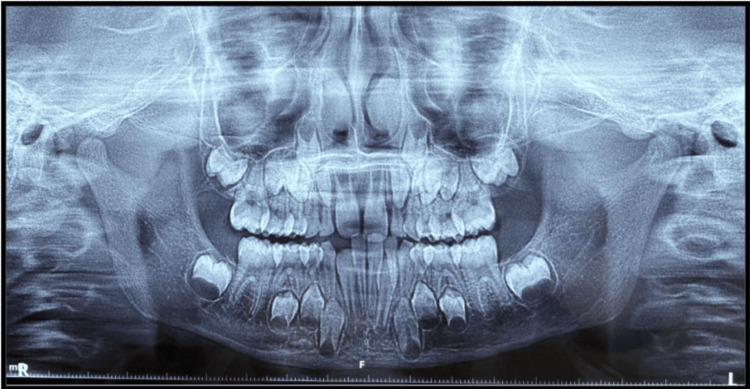
Panoramic radiograph image of case 2 patient's teeth The image shows no evidence of pulpal calcifications.

Magnetic resonance imaging (MRI) of the brain revealed multiple subependymal nodules along the lateral ventricular walls (Figure 13), consistent with TSC.

**Figure 13:**
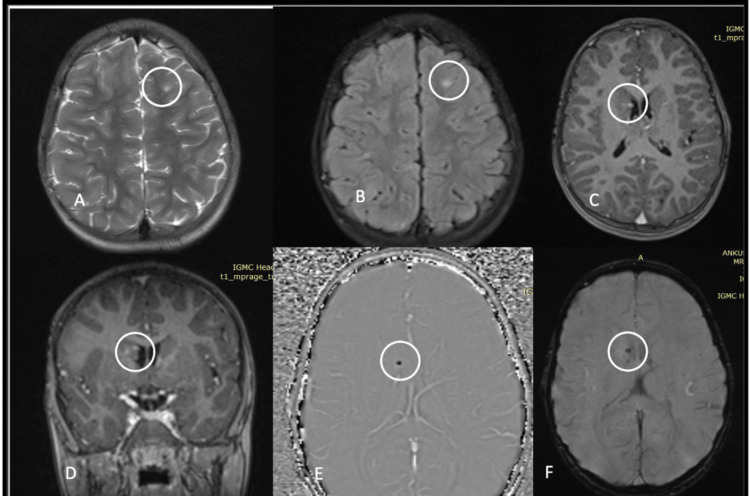
T2-FLAIR MRI (axial) images of case 2 patient's brain The images show a hyperintense lesion in the left frontal lobe (A, B); T1 hyperintense (C, D); and a calcified subependymal nodule (E, F).

Treatment and Management

Given the limited oral involvement, management was entirely conservative and preventive. The patient and his parents received age-appropriate oral hygiene instruction, demonstration of supervised brushing technique, and dietary counseling. Following professional prophylaxis, topical 5% sodium fluoride varnish was applied, and resin-based pit-and-fissure sealant was placed on the affected teeth (11 and 21) to seal the enamel defects. No restorative or surgical intervention was required at presentation.

Multidisciplinary referrals were arranged in parallel and mirrored those for his sister, in accordance with the international TSC surveillance protocol [8]. These included pediatric neurology for evaluation of the subependymal nodules and seizure-risk monitoring; dermatology for management of the facial angiofibromas, ungual fibromas, and confetti macules; ophthalmology for retinal examination; and abdominal imaging to exclude renal angiomyolipomas. Genetic counseling was offered to the family alongside the elder sibling’s consultation.

Response and Follow-up

All preventive procedures were well tolerated. At the three-month review, the sealants were intact, plaque control was satisfactory, and there were no new oral findings. The patient has been enrolled in the same long-term dental and systemic recall program as his sister, with continued biannual dental review and structured multidisciplinary surveillance.

## Discussion

TSC demonstrates autosomal dominant inheritance with marked intrafamilial variability. The present family clearly illustrates this phenomenon. Although all three affected members share the same underlying genetic predisposition, their clinical phenotypes diverge considerably. The father’s disease has been dominated by seizure activity, whereas both children remain neurologically asymptomatic despite imaging evidence of subependymal nodules. Such intrafamilial heterogeneity has been attributed to several mechanisms, including the age-dependent emergence of clinical features, somatic second-hit mutations producing tissue-specific lesions, and modifier genes that influence disease severity [8]. The current asymptomatic neurological status of both siblings does not exclude future progression and reinforces the need for sustained multidisciplinary follow-up.

Orofacial manifestations occupy an increasingly recognized position within the diagnostic framework of TSC. According to the 2021 updated consensus criteria, the presence of three or more dental enamel pits constitutes a major diagnostic feature, while two or more intraoral fibromas similarly qualify as a major criterion [7,8]. The elder sibling in the present report fulfilled both of these dental criteria, whereas the younger sibling met only the enamel-pit criterion. This intrafamilial discordance in oral expression mirrors observations in earlier reports [2,5] and underscores the discriminatory value of a meticulous oral examination.

A noteworthy observation in the elder sibling was the presence of multiple pulpal calcifications. Pulpal calcifications are not currently incorporated into the established TSC diagnostic criteria, and reports of their occurrence in this disorder remain scarce. Whether such findings represent a previously underrecognized dental feature of TSC or an incidental coincidence cannot be resolved from a single case; nevertheless, the observation may warrant systematic evaluation in larger cohorts. The shagreen patch identified in the elder sibling was located over the facial region, an uncommon site, as this lesion is more typically encountered in the lumbosacral area. Reports of facial or other extralumbosacral involvement are sparse, and this presentation further emphasizes the phenotypic breadth of TSC and the importance of inspecting the entire integument when the diagnosis is suspected.

Collectively, these observations highlight the contributory role of dental practitioners in the early identification of TSC. Because oral and dental features may sometimes precede or accompany the more widely recognized systemic manifestations, dentists who encounter such findings should pursue a comprehensive evaluation and refer affected individuals for multidisciplinary care, including pediatric, neurological, dermatological, and genetic assessment [4,7].

## Conclusions

Tuberous sclerosis complex demonstrates marked intrafamilial phenotypic variability, even among affected members of the same family. The present report highlights differences in systemic, cutaneous, and oral manifestations between two siblings, emphasizing the variable expression of the disease and the importance of comprehensive clinical evaluation and long-term surveillance. Oral findings such as enamel pits and gingival fibromas may serve as valuable diagnostic indicators, particularly in individuals with minimal or absent neurological manifestations. The presence of pulpal calcifications in one sibling, although not currently recognized as a diagnostic feature and infrequently reported in the literature, may represent an incidental or underreported finding. Furthermore, the absence of seizures in the children at present does not preclude their future development, underscoring the age-dependent nature of disease expression in TSC. Early recognition of characteristic oral findings by dental professionals can facilitate timely diagnosis, appropriate multidisciplinary management, and ongoing patient monitoring.
